# One-pot synthesis of quinazolinone heterocyclic compounds using functionalized SBA-15 with natural material ellagic acid as a novel nanocatalyst

**DOI:** 10.1038/s41598-024-61803-y

**Published:** 2024-05-16

**Authors:** Nazanin Mohassel Yazdi, Mohammad Reza Naimi-Jamal

**Affiliations:** Research Laboratory of Green Organic Synthesis & Polymers, Department of Chemistry, Iran University of Science and Technology, P.O. Box 16846–13114, Tehran, Iran

**Keywords:** Heterogeneous catalysis, Synthetic chemistry methodology

## Abstract

The nanoporous compound SBA-15 was functionalized using (3-aminopropyl)trimethoxysilane (APTES). Then the obtained product was modified with ellagic acid (ELA), a bioactive polyphenolic compound. The structure of the prepared nanoporous composition SBA-15@ELA was extensively characterized and confirmed by various techniques, such as Fourier-transform infrared (FT-IR) spectroscopy, Energy dispersive X-ray (EDX) elemental analysis, scanning electron microscopy (SEM), thermogravimetric analysis (TGA), X-ray diffraction (XRD), transmission electron microscopy (TEM) and N_2_ adsorption–desorption isotherms (BET). The novel, recoverable, heterogenous SBA-15@ELA nanoporous compound was used to investigate its catalytic effect in the synthesis of 4-oxo-quinazoline derivatives (19 examples) with high yields (78–96%), as an important class of nitrogen-containing heterocyclic compounds. The use of an inexpensive mesoporous catalyst with a high surface area, along with easy recovery by simple filtration are among the advantages of this catalysis research work. The catalyst has been used in at least 6 consecutive runs without a significant loss of its activity.

## Introduction

**Figure 1:**
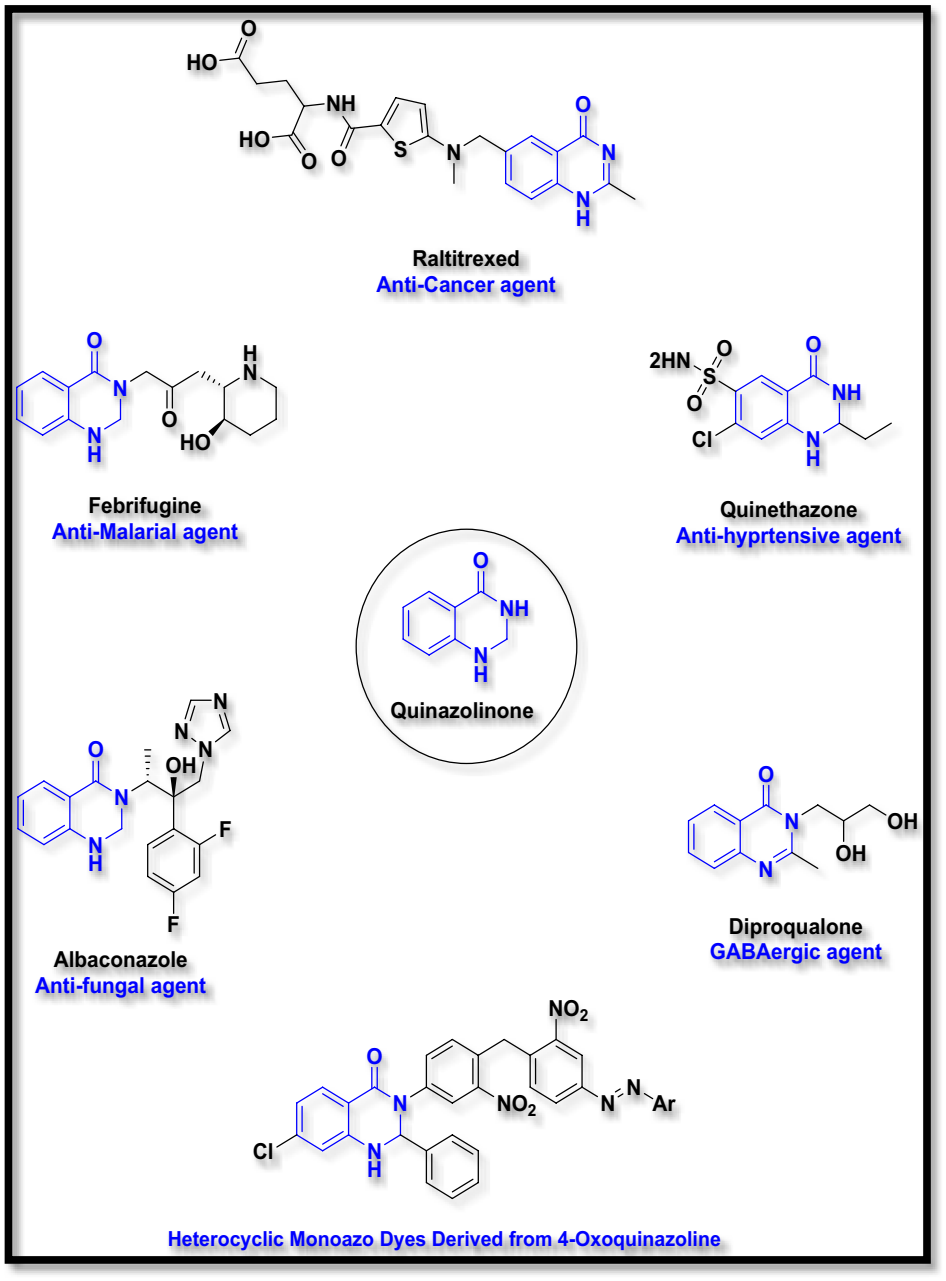
Biological and non-biological compounds with 4-oxoquinazoline skeleton.

In recent years, among nitrogen-containing fused heterocyclic rings, quinazoline and quinazolinone structures have attracted great interest in medicinal chemistry for the development of new drugs^1^. They display diverse biological activities such as anticancer^2^, anticonvulsant^3,4^, anti-malarial^5^, antidiabetic^6^, anticholinesterase^7^, antifungal^8,9^, and kinase inhibitory activities^10^. Many synthetic and natural compounds contain quinazoline rings in their structure. In Fig. 1, some of the structures containing this skeleton with their usage have been shown^1^. For example, *Febrifugine* was first extracted from the *Dicora febifuga* plant in China in 1947 and used as an anti-malarial agent^5^.

In addition to wide biological and biomedical^11^ applications, these structures have other applications in industries. Many researchers and dye industry researchers have been trying to design new and active dyeing structures for profitable commercial uses such as dyeing silk, wool, wood, etc.^12^. In the meantime, fused heterocyclic compounds such as 4-oxo-quinazoline and their derivatives have been able to occupy a special position^13^. In addition to having a wide range of colors from yellow to red, the compounds containing quinazoline skeleton; create a certain uniformity and shine on the used surface^14^.

During the research conducted by various researchers in recent years, it was found that some quinazolinone derivatives show good luminescence properties. With excellent biocompatibility, low toxicity, and high efficiency, these compounds have become promising candidates for use as fluorescent probes, biological imaging reagents, and luminescent materials^15^. Certain quinazolines have the potential to serve as building blocks for the creation of biologically active compounds, including several alkaloids e.g. *Leutonin B* and *E*, *Bouchardatine* and *8-Norrutaecarpine*^16^.

There are many reports available on the design of quinazoline/quinazolinone-based structures^17^. Common synthetic methods include Aza-Wittig method (via synthesis of iminophosphorane intermediates)^18,19^, synthesis in microwave oven^20^, synthesis in solid phase^21^, use of organometallic reagents^22^, synthesis through ring closing reactions^23^, synthesis with nitriles^24^, synthesis with guanidium and imidate salts etc., which in all the mentioned methods anthranilic acid derivatives have been used as starting materials^17^. Meanwhile, the use of isatoic anhydride as a raw material has been one of the favorite methods of researchers to prepare these derivatives^25,26^. Another method for the synthesis of these heterocyclic rings, which is more efficient than the previously mentioned methods, is the use of multi-component reactions. Using various substances such as ammonium salts, aromatic first type amine derivatives as the second component and ortho-esters^27^, aromatic aldehydes or cyclic ketones^28^ as the third component led to the product 4-oxoquinazolines. From the interaction of isatoic anhydride and hydrazine hydrate, the product 2-aminobenzohydrazide is formed, which in case of interaction with ortho esters, N-aminoquinazoline is obtained.

Porous materials are among the most widely used materials in various fields in recent years^29,30^. These compounds are of researchers’ interest due to their advantages such as large surface area, uniform pore size, well-defined pores, and potential for functionalization with several chemicals^31^. According to the IUPAC classification, SBA-15 composites belong to the mesoporous (2-50nm) category, and the size of their uniform hexagonal pores is about 30 nm. This compound was first synthesized in acidic media to produce highly ordered, two-dimensional hexagonal silica-block copolymer mesophases in 1992 by Stucky and co-workers at the University of California^32^. SBA-15 is used in a wide range of fields, such as drug delivery^33^, as a catalyst^31^, water treatment^34,35^, bioimaging, biomedical applications, therapeutic/diagnostic applications, and also analytical chemistry, due to the "structure relationship"—Exceptional performance. The use of functionalized SBA-15 as a catalyst in multicomponent reactions is of great interest among organic and inorganic chemists. Modifying the surface of SBA-15 with various synthetic and natural compounds, as well as doping different metals such as Cu or Pd creates various catalytic structures^36,37^. *Heravi* and co-workers accelerated the A^3^-coupling reaction by making a new heterogeneous catalyst based on SBA-15 and doping Cu on it.

Also, manufacturing new composites which are mainly composed of SBA-15 and MCM-41 as a mesoporous component^38^, iron oxide MNPs (magnetic nanoparticles) as magnetic parts^39,40^, and modified natural polymers have been reported in recent years^41,42^.

**Figure 2:**
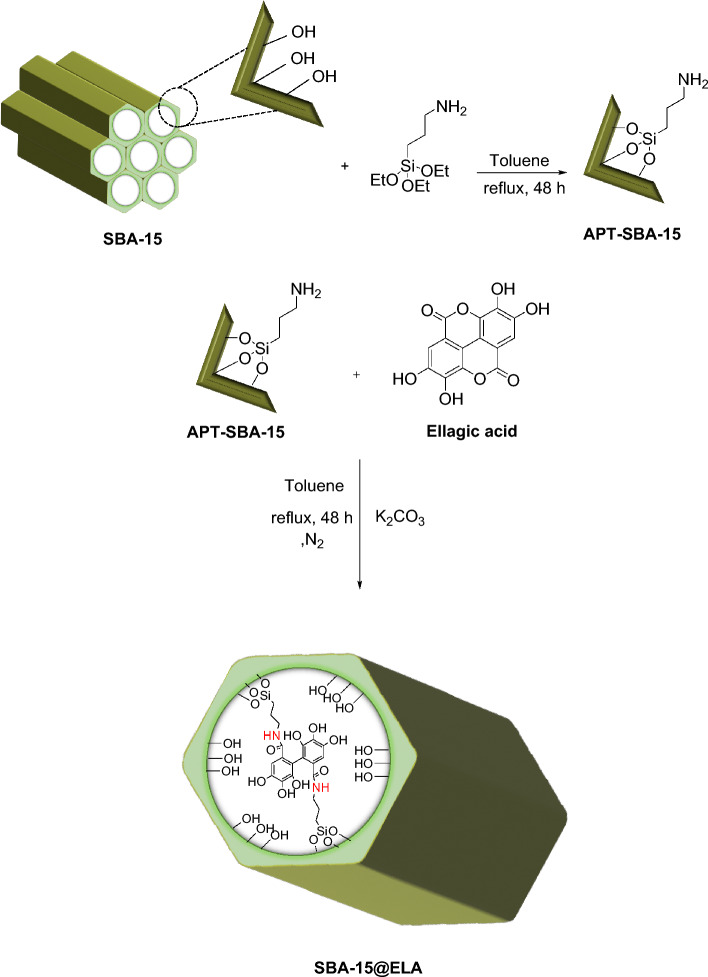
Schematic preparation route of SBA-15@ELA catalytic system.

Herein, we introduce a new catalytic system for the synthesis of 4-oxo-dihydroquinazolinone derivatives. In the fabrication of this novel mesoporous nanocomposite, formulated as “SBA-15@ELA”, at first SBA-15 was prepared with Stucky's method, then SBA-15 was modified with APTES to obtain SBA-15–NH_2_^43^. An important step in preparing the final composite is the interaction of -NH_2_ from APTES and carbonyl from a cyclic ester of Ellagic acid to form an amide bond through a nucleophilic substitution reaction (Fig. 2). The fabricated nanocomposite has unique physicochemical properties because of its organic–inorganic structure.

**Figure 3:**
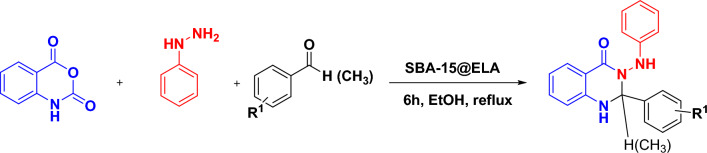
Synthesis of 4-oxo-dihydroquinazolinone derivatives in the presence of SBA-15@ELA nanocatalyst.

After characterizing and proving the structure of SBA-15@ELA with different techniques, to investigate the catalytic properties of this heterogeneous nanoporous composite, we used it in a one-pot multicomponent reaction for the synthesis of 4-oxo-dihydroquinazolinone compounds (Fig. 3).

## Material and methods

### Reagents and instrumentation

All raw materials and solvents used in this work were purchased from reputable companies such as Merck and Sigma-Aldrich and were used without any purification. The Synthesis of SBA-15 is according to the traditional recipe of Stucky^32^. All the reactions were monitored by TLC (Thin-Layer Chromatography) on pre-coated silica gel plates (0.25 mm) and visualized by UV–Vis light at 254 nm. The melting points of the prepared derivatives were measured by an Electrothermal 9100 apparatus, which was reported without any correction. Elemental analysis was provided by EDX analysis, which was recorded by TESCAN4992. The FT-IR spectra were recorded in the range of 400–4000 cm^–1^ using the Perkin Elmer 1720-X spectrometer by using KBr pellets. The N_2_ adsorption–desorption isotherms are measured by BELSORP-mini II. The morphology of the synthesized nanocomposite was studied by SEM using MIRA2 TESCAN instrument. The TGA of the prepared nanocomposite was obtained by an STD Q600. The XRD measurements were recorded with the Rigaku Ultima IV.

### Catalyst preparation

#### Synthesis of SBA-15

The composition of SBA-15 was prepared using the method reported by Stuckey et al. Briefly; 120 g of hydrochloric acid 2 M and 4 g of Pluronic P123 were poured into a 500 ml round-bottom flask containing 30 g of distilled water. The temperature of the reaction mixture was set at 40 °C and was being vigorously stirred. 8.5 g of TEOS (Tetraethyl orthosilicate) was added dropwise to the reaction mixture and stirring was continued for 8 h. The resulting mixture was transferred to a stainless-steel autoclave with Teflon bottle sealed, which was then heated to 100 °C for 2 days in an oven without any stirring. The composition of the gel is P123:HCl:H_2_O:TEOS with molar ratios of 0.0168:5.854:162.681:1. After cooling the mixture to ambient temperature, the reaction product was filtered off and washed with distilled water three times and dried for 24 h at 60 °C. The resulting white solid was then calcinated at 550 °C for 6 h.

#### Synthesis of SBA-15–NH_2_^44^

One gram of SBA-15 synthesized in the previous step was poured into 30.0 mL of toluene in a 100 mL flask. An ultrasonic bath was used to thoroughly disperse the reaction mixture. The mixture was allowed to stir at 120 °C under reflux for 48 h under N_2_ condition after adding 2.0 mL of APTES. The precipitate was washed several times with Distilled water and ethanol and placed in an oven at 60 °C for 12 h to dry.

#### Synthesis of SBA-15@ELA

At first, an ultrasonic bath was used to thoroughly disperse the reaction mixture. One gram of SBA-15–NH_2_ along with 50 mL of toluene solvent was poured into a round-bottom flask and let to stir for half an hour at 120 °C until it spread evenly. Three mmol of K_2_CO_3_ as a base and 3 mmol of ellagic acid (equivalent to 0.9 g) were added to the stirring suspension for 48 h at reflux temperature (120 °C) under inert N_2_ gas. After 48 h, the precipitate was filtered off and washed with ethanol and acetone several times to completely remove toluene and excess ellagic acid. The final product was dried in a vacuum oven at 70 °C for 10 h.

### General procedure for preparation of 2-aryl-3-(arylamino)-2,3-dihydroquinazolin-4(1H)-ones

4-Oxo-dihydroquinazolinones **4a–t** were synthesized through a one-pot three-component reaction of isatoic anhydride (ISA, **1**), phenylhydrazine (**2**) and aromatic aldehydes or acetophenone derivatives (**3a–t**). Isatoic anhydride (1 mmol, 0.163 g), and phenylhydrazine (1.1 mmol, 0.118 g) were placed in a round-bottomed flask and SBA-15@ELA nanocatalyst were added. Ethanol (5 mL) was added, and the mixture was heated for an hour under reflux. When the reaction was completed (no more ISA was detected by TLC; n-hexane–EtOAc, 3:1), the aldehyde or ketone derivative (1 mmol) was added to the above mixture. The reaction was stirred and heated under reflux for 6 h. After completion of the reaction (TLC; n-hexane–EtOAc, 3:1), the catalyst was separated from the reaction mixture with filter paper, then the solvent was evaporated under reduced pressure, and EtOH-H_2_O (5:5 mL) was added slowly and mixture was stirred for an hour at R.T. The precipitate was filtered off and washed with cold H_2_O.

## Results

### Characterization of catalysts

After the preparation of the SBA-15@ELA, different techniques were used to characterize its structure.

### FT-IR

**Figure 4:**
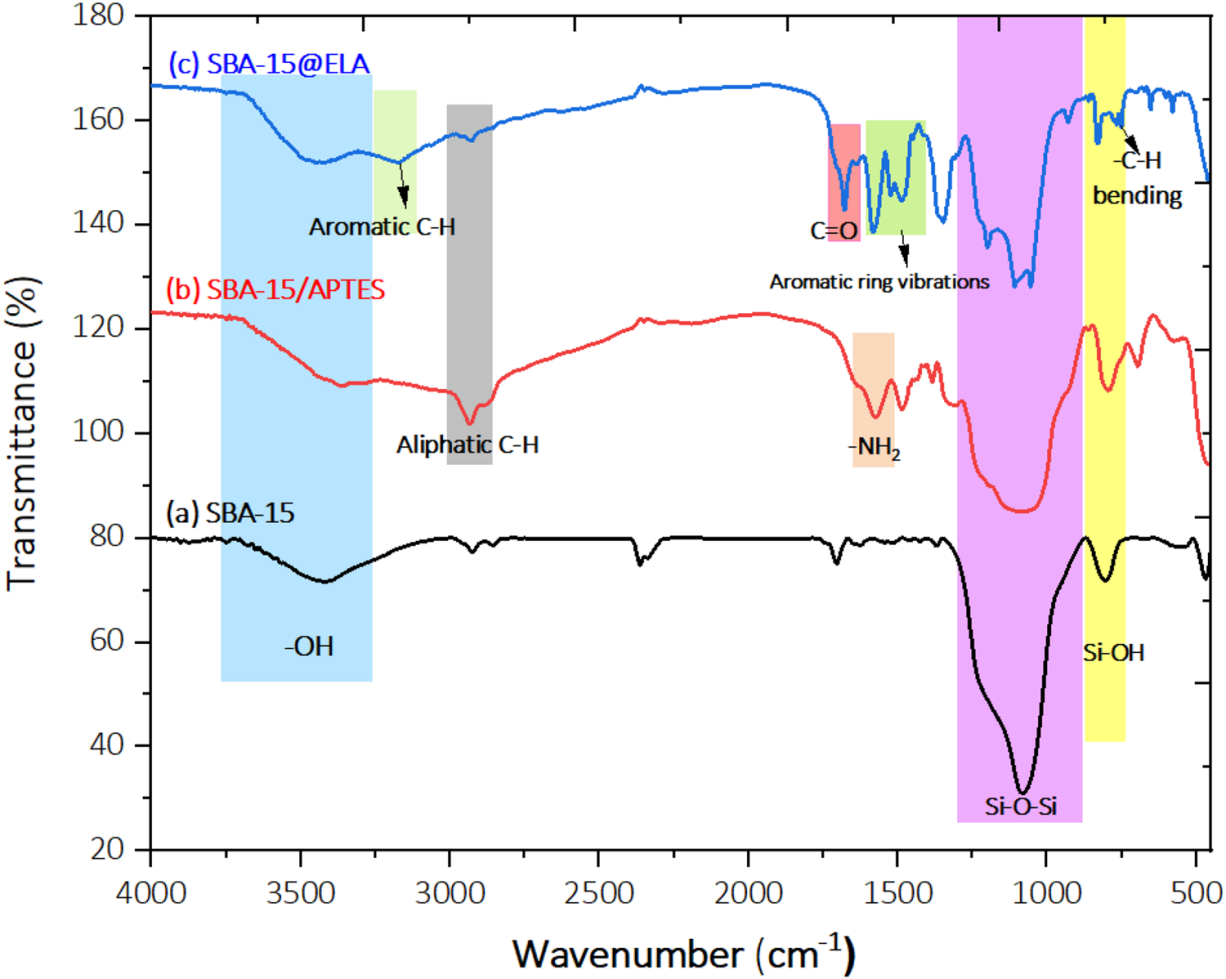
FT-IR spectra of: (**a**) SBA-15, (**b**) SBA-15/APTES, (**c**) SBA-15@ELA.

FT-IR analysis was used to check the presence of functional groups in the samples prepared at each stage of final nanocomposite preparation. The silica network has characteristic absorption bands at about 472 cm^−1^ (bending vibration of Si–O–Si), about 792–970 cm^−1^ (stretching vibration of Si–OH), about 1092 cm^−1^ (asymmetric stretching vibration of Si–O–Si), and about 3440 cm^−1^ (stretching vibration of Hydroxyl groups)^45,46^ (Fig. 4a). In addition to the peaks mentioned, the peak of 2930 cm^−1^ indicates stretching vibrations of aliphatic C-H bonds of the propyl chains. Also, the peak at 1575 cm^−1^ proves the presence of C-NH_2_ functional group (Fig. 4b)^47^. The presence of an obvious peak at 3171 cm^−1^ indicates the presence of aromatic C-H bonds in SBA-15@ELA. The band at 1680 cm^−1^ is attributed to the carbonyl of the amide group. The bands observed in the range, 1679–1460 cm^−1^ are due to aromatic ring vibrations^48^. In addition, the band at 751 cm^−1^ is assigned to aromatic C-H bending vibration and the broad peak of the region of 1092 cm^−1^ Si–O–Si bonds can still be seen (Fig. 4c).

### EDX

**Figure 5:**
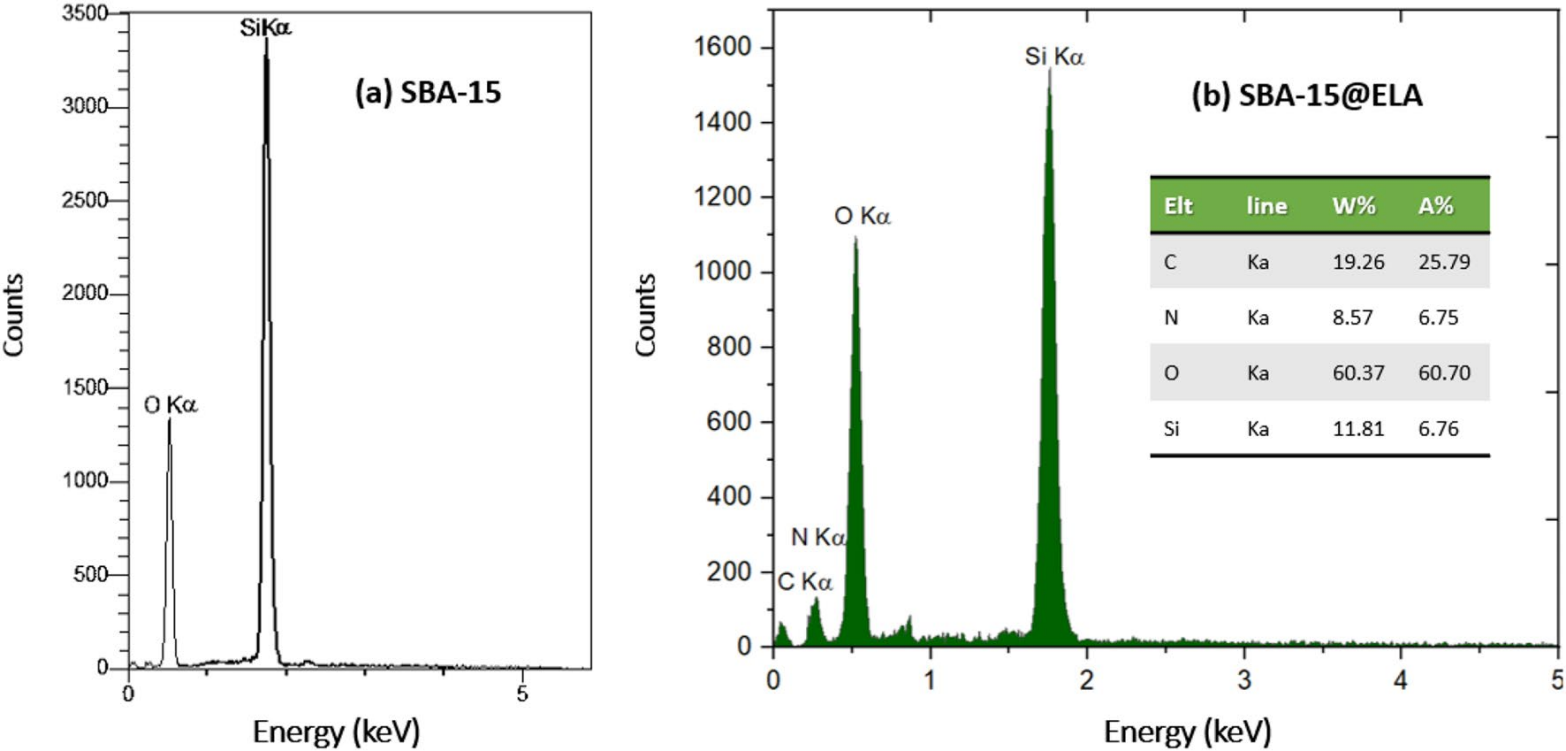
EDX Analysis of (**a**) SBA-15, (**b**) SBA-15@ELA.

Energy Dispersive X-Ray Analysis (EDAX) was used to identify the elemental composition or chemical characterization. As is shown in the EDX spectrum of Fig. 5a, O and Si are the elemental constituent of SBA-15, while in SBA-15@ELA Fig. 5b, the presence of C, O, Si, and N is obvious. The presence of carbon and nitrogen alongside oxygen and silicon suggests that ellagic acid, which contains these elements, has been successfully introduced to the SBA-15 substrate.

### FESEM

**Figure 6:**
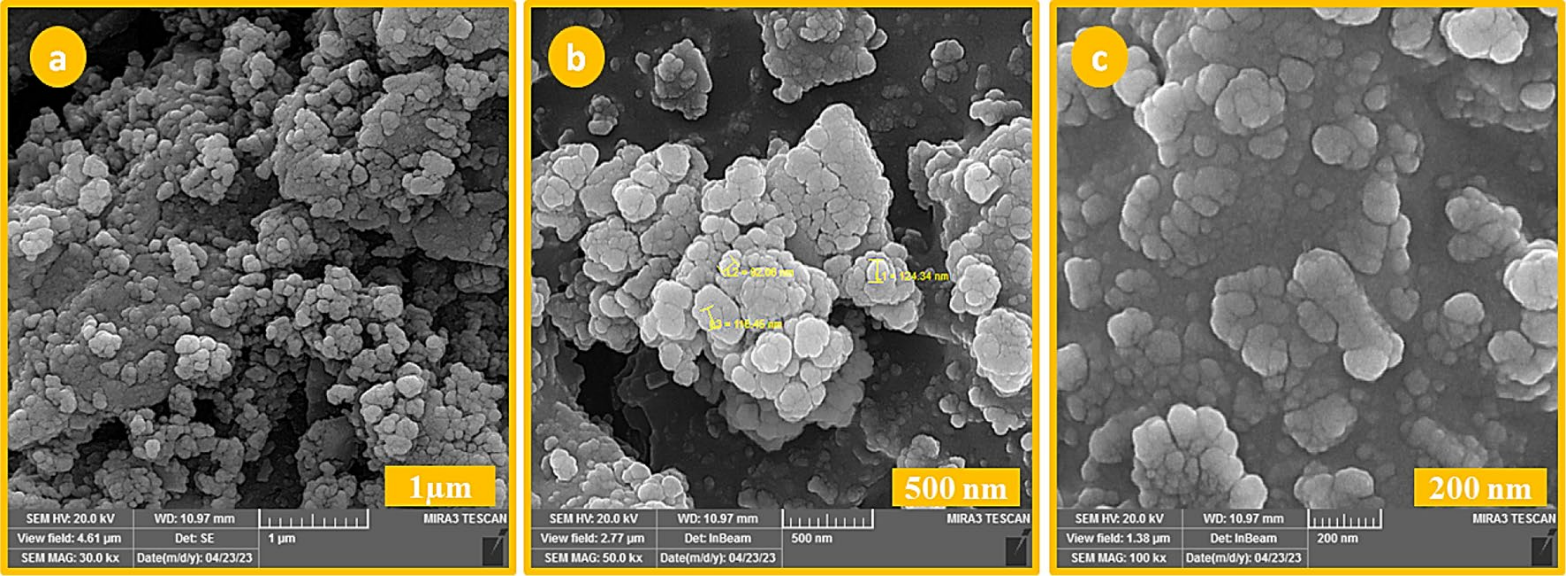
FESEM images of SBA-15@ELA structure.

Field emission scanning electron microscopy was used to observe the surface morphology, particle size distribution, and aggregate state of particles in the prepared SBA-15@ELA structure. As shown in Fig. 6, FESEM images of SBA-15@ELA structure are presented in three scales: 1 µm, 500 nm, and 200 nm.

**Figure 7:**
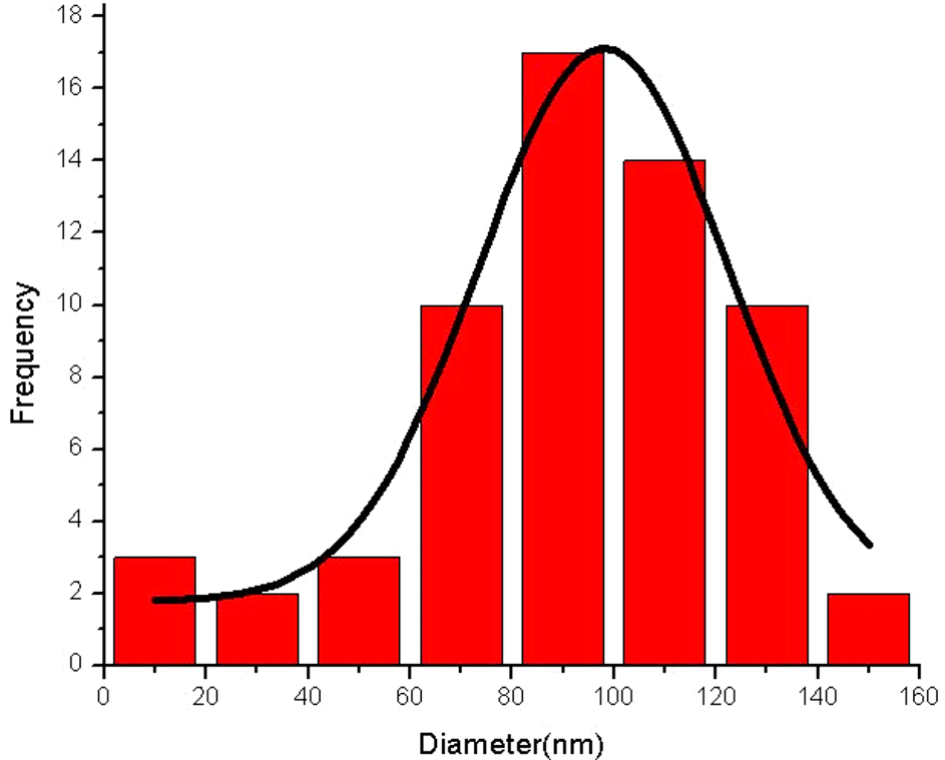
Size Distributions of SBA-15@ELA nanoparticles.

The porous and sheet-like structure of SBA-15@ELA structure is shown in the pictures. By measuring the size of the particles using Digimizer software, and drawing the distribution diagram (Fig. 7), it was found that most of the particles are around 80–90 nm.

### TEM

**Figure 8:**
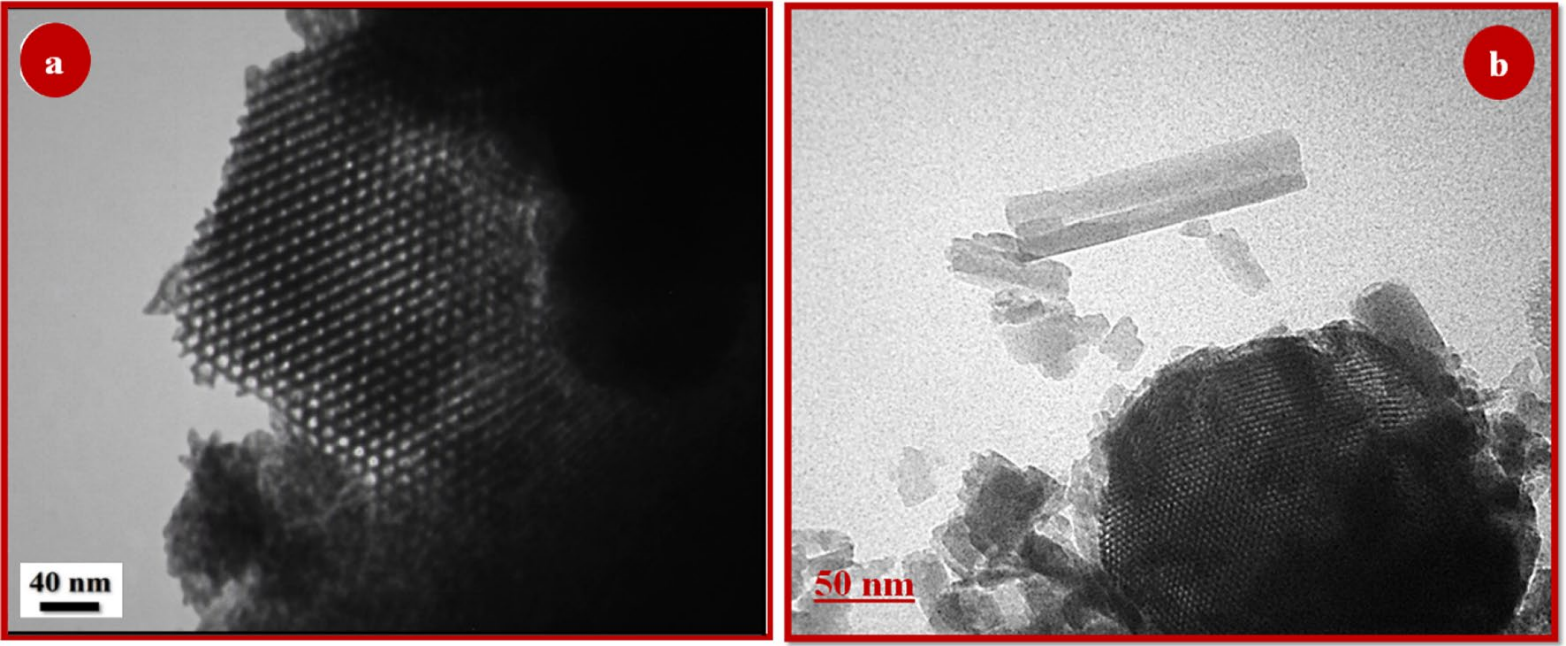
TEM images of (**a**) SBA-15 and (**b**) SBA-15@ELA structures.

To further investigate the surface morphology and structure, Transmission electron microscopy images of the SBA-15 and SBA-15@ellajic acid structures are shown in Fig. 8. In the Fig. 8a on the left image, the regular two-dimensional hexagonal honeycomb structure of the SBA-15 are clearly visible. In the catalyst SBA-15@ELA (Fig. 8b), voids are observed resembling parallel channels akin to SBA-15^35^, affirming that the integrity of the pore structure remained unaffected during the functionalization process. For both images, it's important to note that the absence of visible channels doesn't necessarily imply that the structure isn't there—it could also be a result of the sample's orientation relative to the microscope's line of sight. Furthermore, the dark and light contrast in TEM images can be influenced by the thickness, density, and composition of the material.

### XRD

**Figure 9:**
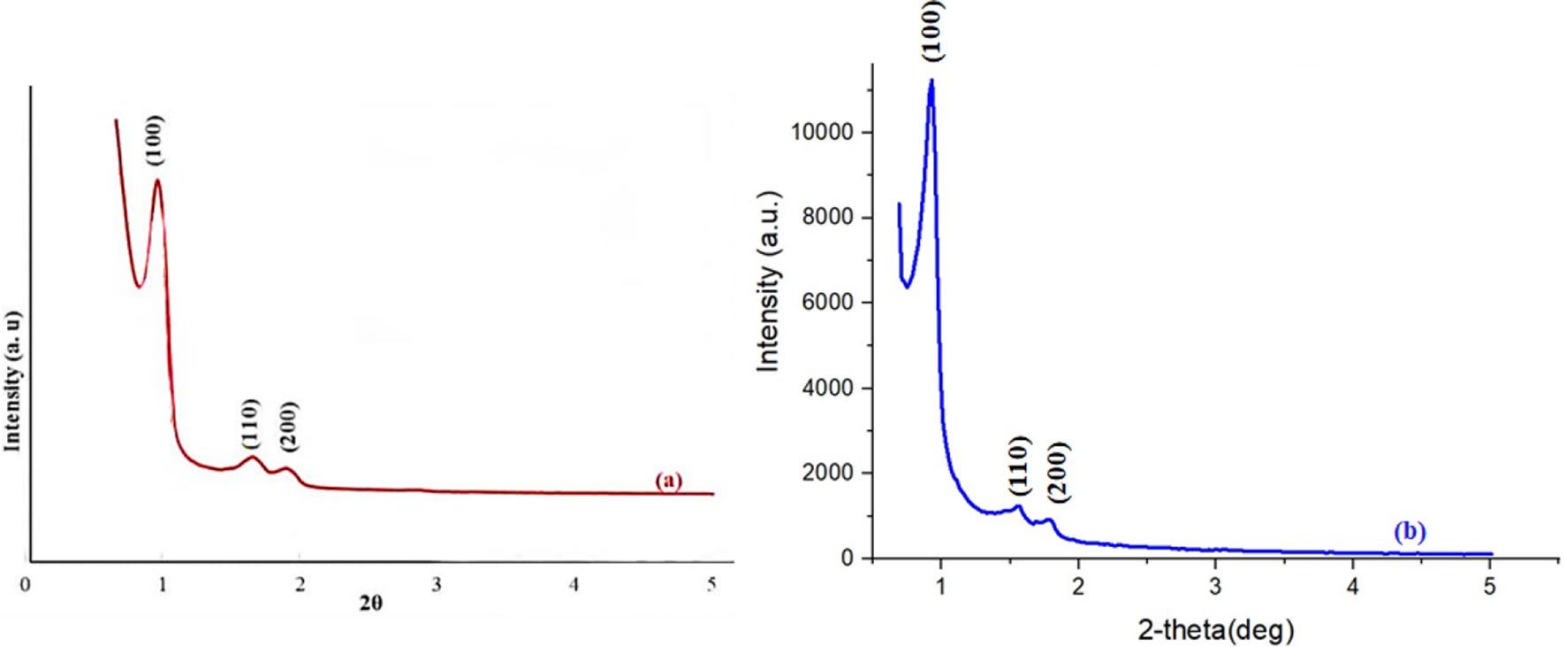
Low-angle XRD patterns of (**a**) SBA-15, (**b**) SBA-15@ELA.

**Figure 10:**
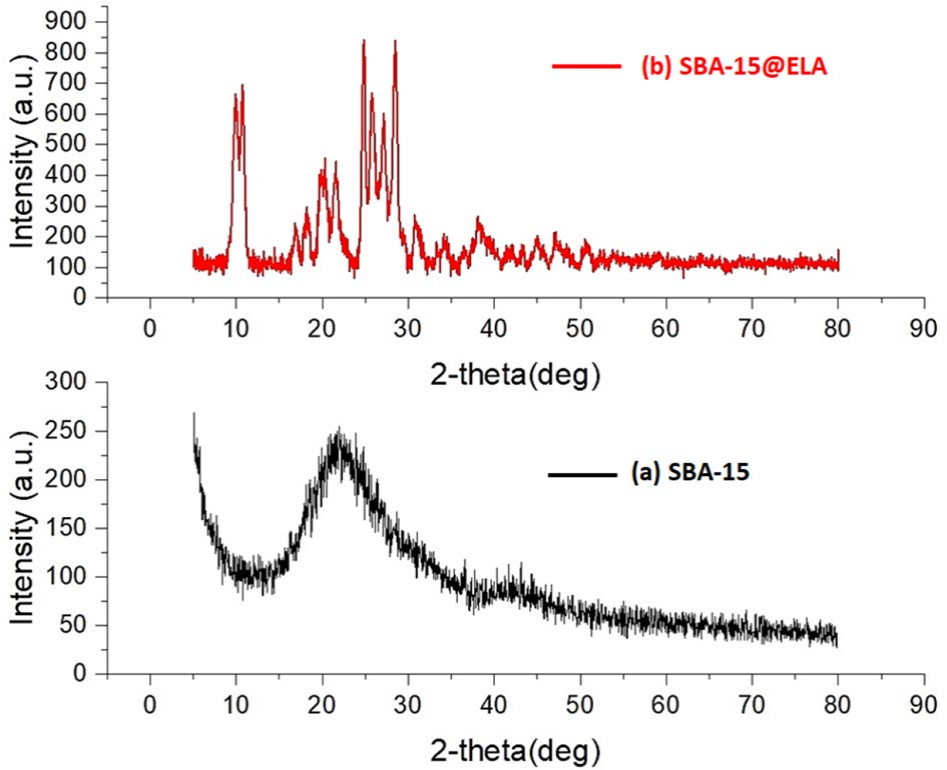
High-angle XRD patterns of (**a**) SBA-15, (**b**) SBA-15@ELA.

To investigate the crystalline structure of the SBA-15@ELA, the analysis of X-ray diffraction patterns was used in both wide and low-angle modes. According to Fig. 9a, the X-ray diffraction pattern in the range of 0 to 5 degrees (Low-angle XRD) for the structure of SBA-15 and Fig. 9b for the structure of SBA-15@ELA indicated that one high intensity peak at 2Ө: 0.91 and two small peaks at 2Ө: 1.58 and 1.82 corresponding to (1 0 0), (1 1 0) and (2 0 0) planes, respectively^44^. They are typical hexagonally structured SBA-15 with highly ordered mesoporous channels. Furthermore, these peaks indicate the structural integrity maintained throughout the surface functionalization steps, highlighting the retention of the distinctive features of the initial mesoporous SBA-15 composition in the SBA-15@ELA. The decrease in diffraction intensity observed in the SBA-15@ELA composition is attributed to the incorporation of organic groups on the surface. As the diffraction peaks remain unchanged towards higher angles, it suggests that the pores have not undergone closure. In Fig. 10a, the diffractogram of SBA-15 shows a broad distinctive peak in the range of 2ɵ: 20°–30° attributed to the amorphous SiO_2_ character^49^. The observed diffraction peaks for SBA-15@ELA (Fig. 10b) were at 2ɵ values of 10.8, 15.8, 17.3, 21.1, 22.8, 24.2, 24.7, 25.4, 26.7, 28.5, 29.7, 32.7 and 34.8, related to the addition of organic part^48^. These peaks correspond to the crystalline nature of the organic part (ellagic acid) that has been added to the SBA-15 framework.

### BET analysis

**Figure 11:**
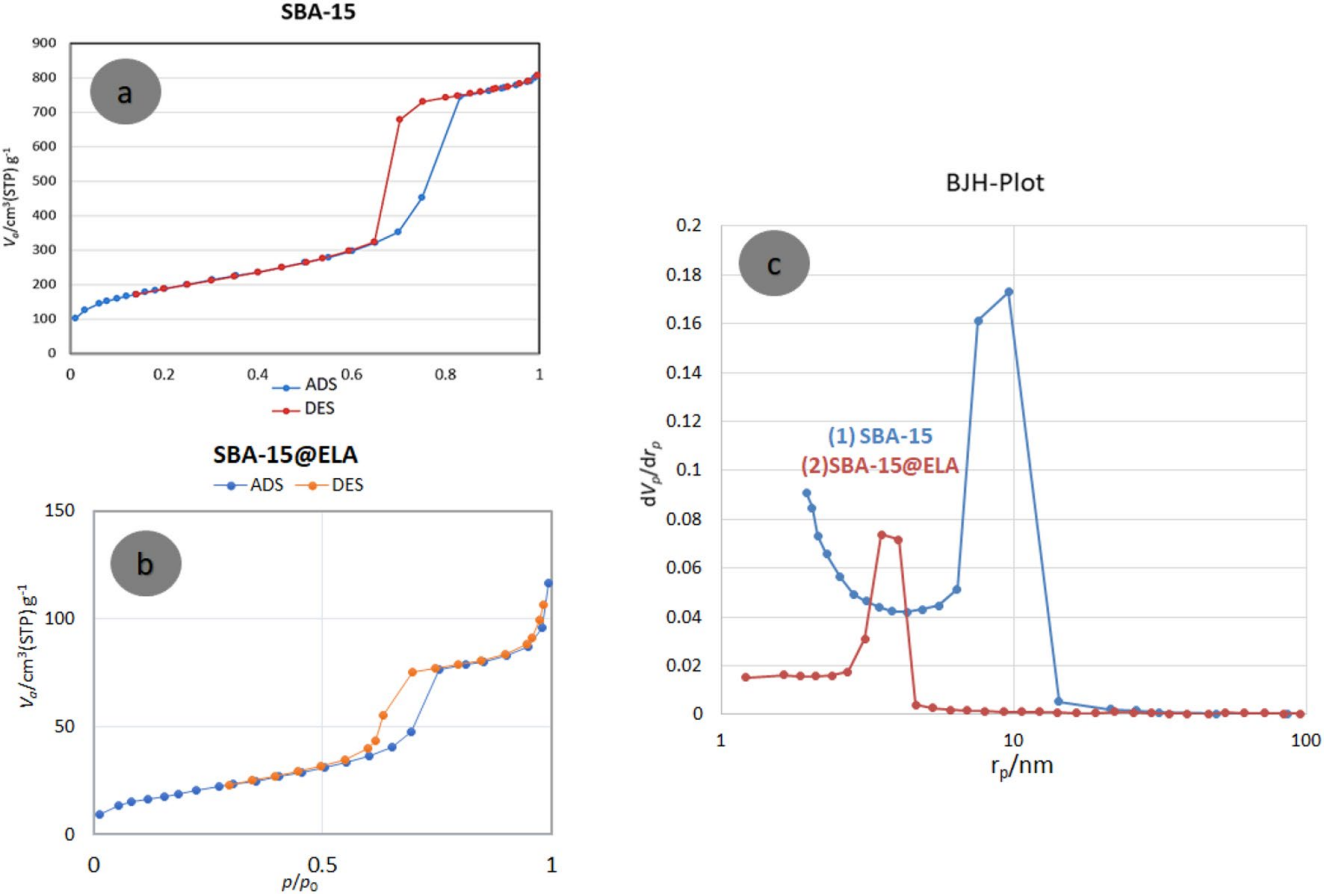
N_2_ adsorption–desorption isotherms of (**a**) SBA-15, (**b**) SBA-15@ELA and, (**c**) the pore size distribution curve of SBA-15@ELA.

The N_2_ adsorption–desorption isotherms of SBA-15 and SBA-15@ELA are presented in Fig. 11a and b. The isotherms of both compounds were classified as type IV, characteristic of mesoporous materials. The sharp capillary condensation of N_2_ into the mesoporous channels at high relative pressure and H1 hysteresis loop based on standard IUPAC categories, indicates the presence of large channel-like pore structures. The surface area of the neat SBA-15 (686.9 m^2^ g^−1^) is more than the surface area of its functionalized structure (Table 1). As shown in Fig. 11a and b this result can be attributed to the blocking of a part of SBA-15 pores by APTES as well as ellagic acid and the changes caused by it, which has reduced the available surface for gas absorption. The pore-size distribution has its maximum at 3.53 nm, as shown in BJH pore size distribution curve of SBA-15@ELA in Fig. 11c.

**Table 1:** Surface area, pore volume and pore diameter of SBA-15 and SBA-15@ELA.

Sample	Surface area (m^2^ g^−1^)	Pore volume (cm^3^ g^−1^)	Pore size (nm)
SBA-15	686.90	1.28	7.35
SBA-15@ELA	90.21	0.17	3.53

#### TGA

**Figure 12:**
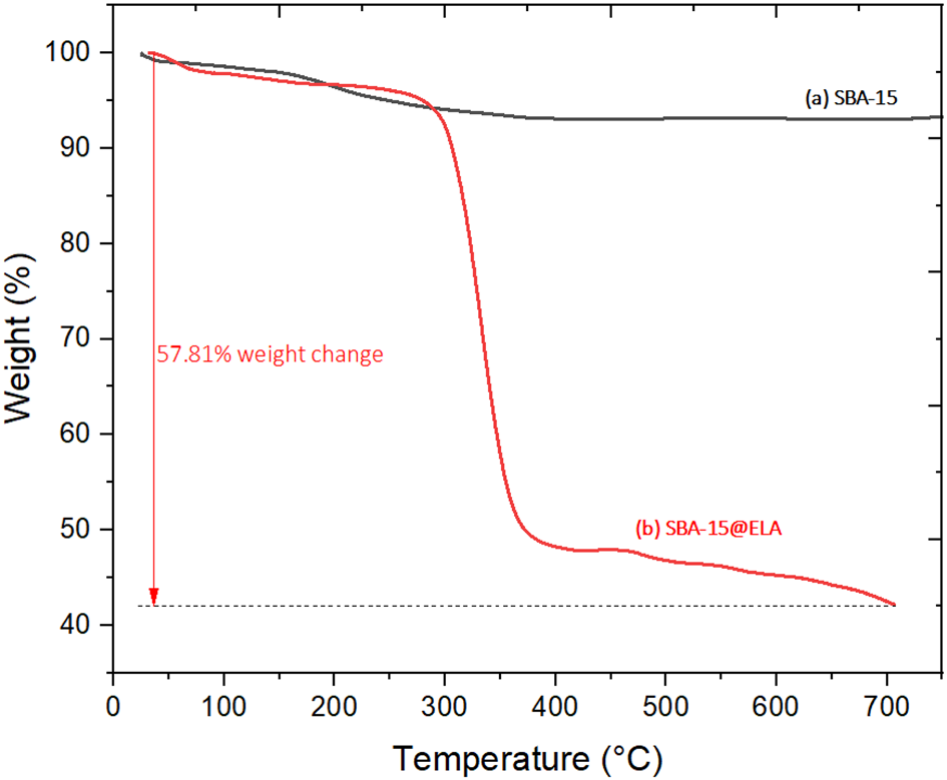
TGA analysis of (**a**) SBA-15 and (**b**) SBA-15@ELA.

The thermogravimetric analysis (TGA) curves of (a) SBA-15 and (b) SBA-15@ELA have been shown in Fig. 12. The black curve (a) for pure SBA-15 remains relatively stable across the entire temperature range, showing no significant weight loss. This indicates that SBA-15 is thermally stable up to 700°C. The red curve (b) belongs to SBA-15@ELA. Weight loss at temperatures less than 150 °C can be attributed to eliminating adsorbed water and other solvents (estimated 10%). It seems that 40% of the structure was decomposed and, major weight loss within the range of 300–360 °C was due to the degradation of organic moieties. The TGA data showed that SBA-15@ELA decomposes at a lower temperature range than pure SBA-15, which is expected as organic materials typically decompose at lower temperatures than inorganic materials like silica. The large weight loss in the SBA-15@ELA sample explains the presence and subsequent breakdown of the organic moieties attached to the SBA-15, confirming the successful functionalization of SBA-15 with ellagic acid.

### Application of catalyst in the synthesis of 4-oxo-dihydroquinazolinone

To investigate the catalytic activity of the proven structure of SBA-15@ELA, this compound was used in the synthesis of 4-oxo-dihydroquinazolinone derivatives. To optimize and obtain the best path, different experimental conditions such as temperature, solvent, amount of catalyst, and type of catalyst were investigated in a model three-component one-pot reaction. To achieve this goal, the preparation of product **4a** was selected as a model reaction. As stated in the table, at first a reaction was tested in H_2_O as a solvent and without a catalyst for 24 h without any product (Table 2, Entry 1). To check the effect of solvent and temperature, the model reaction was tested again without a catalyst and in ethanol solvent and under reflux conditions. But there was no product (Table 2, Entry 2). In the third experiment, the acidic catalyst of *p*-TSA (20 mol%) was selected as an available acidic catalyst and was added to the three-component reaction mixture (Table 2, Entry 3, 25% yield). In the next step, the reaction components were poured without solvent into the ball-milling system and mixed together for 4 h, but no product was formed (Table 2, Entry 4). In the next part, 10 mol% of ellagic acid in EtOH as solvent and reflux condition was used as a catalyst. It was observed that the reaction efficiency of the model increased significantly to 65% (Table 2, Entry 5). It was supposed that SBA-15 before functionalization, can be considered as a porous material that provides an effective surface for the interaction of substrates. To test the effect of SBA-15 in this reaction, 0.01 g SBA-15 was used in toluene as a solvent so that the temperature could be raised to 120 °C (Table 2, Entry 6). The yield was just 65%. To check the catalytic properties of SBA-15@ELA, at first 0.005 g was used in a reaction. The product yield was acceptable (Table 2, Entry 7, 85%). To understand the catalyst effect, more amounts of SBA-15@ELA (0.01 g and 0.02 g) were tested in EtOH and reflux conditions, with 96 and 95% yield, respectively. The results of the tests showed that adding more than 0.01 g of catalyst SBA-15@ELA has no significant effect on increasing the product even with increasing time (Table 2, Entries 7, 8, 9). The model reaction was retested in the presence of the optimized amount of catalyst SBA-15@ELA (0.01 g) in solvent-free condition (ball-milling), at room temperature and for 2.5 h, a favorable yield (81%) was obtained, but it seems that the presence of the green solvent EtOH provides better condition for more interactions and a higher yield is obtained (Table 2, Entry 10). In addition to the tests mentioned in the table, some others were tested in different solvents such as acetonitrile, MeOH, DMF, but finally, the conditions given in (Table 2, Entry 8) were chosen as the optimal conditions for the model reaction. For further investigation, some other tests were performed on the model reaction in solvent-free conditions. In an experiment, a very small amount of EtOH as a solvent was added to the ball-milling jar. The addition of solvent has a small change in the product of the model reaction (Table 2, Entry 11). In another experiment, the reaction was placed on the heater stirrer, and all the reaction conditions were the same as before (like Entry 8), except that no solvent and no heating was added to the reaction mixture. It was found that the Reaction progress is not possible (Table 2, Entry 12). In the last experiment, the reaction mixture was heated without solvent and no product was formed (Table 2, Entry 13).

**Table 2:** Optimizing the multicomponent reaction conditions in the synthesis of 4-oxo- dihydroquinazolinone as model reaction.


Entry	Catalyst	Solvent	Conditions	Time (h)	Yield (%)
1	–	H_2_O	R.T.	24	N.R.
2	–	EtOH	Reflux	12	N.R.
3	*p*-TSA (20 mol%)^a^	EtOH	Reflux	10	25
4	–	Solvent-free^b^	R.T.	4	N.R.
5	ELA (10 mol%)^c^	EtOH	Reflux	24	65
6	SBA-15 (0.01g)	Toluene	120 °C	10	45
7	SBA-15@ELA (0.005g)	EtOH	Reflux	6	85
**8**	**SBA-15@ELA (0.01g)**	**EtOH**	**Reflux**	**6**	**96**
9	SBA-15@ELA (0.02g)	EtOH	Reflux	12	95
10	SBA-15@ELA (0.01g)	Solvent-free^b^	R.T.	2.5	81
11	SBA-15@ELA (0.01g)	EtOH^d^	R.T.	2.5	85
12	SBA-15@ELA (0.01g)	Solvent-free^e^	R.T.	24	N.R.
13	SBA-15@ELA (0.01g)	Solvent-free^e^	70 °C	24	N.R.

Significant values are in bold.

^a^p-toluene sulfonic acid.

^b^with ball-milling.

^c^Ellagic acid.

^d^with ball-milling.

^e^with heater-stirrer in flask.

After optimizing and finding the best amount of catalyst, temperature, and time for the model reaction, **4b** to **4t** derivatives were synthesized with different starting materials (Table 3). The results showed that electron-donating substitutions (**4a**, **4c**, **4k**) increase the yield, and electron-withdrawing substitutions such as -NO_2_ (**4b**) decrease the yield in the products. Also, *-para* substitution in aldehyde substrates with the same substitution type had better performance in the multicomponent reaction (**4c**, **4g**). In some reactions, ketone derivatives such as acetophenones (**4p**, **4s**) and cyclohexanone (**4m**) were used instead of aldehydes. Synthesized spiro products had lower yields than aldehydes.

**Table 3:**
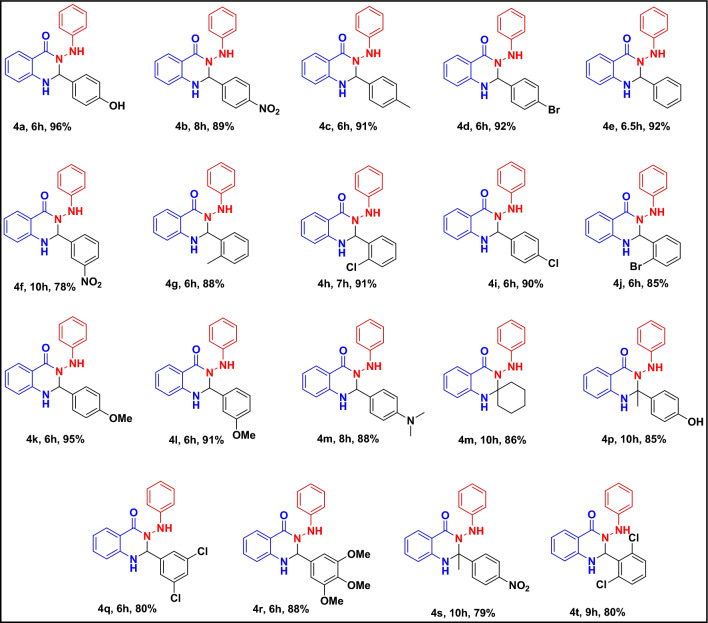
Synthesis yields of different 4-oxo- dihydroquinazolinone derivatives by using SBA-15@ELA catalyst under the optimized conditions.

### Proposal mechanism

As previously reported in various articles, mesoporous compounds with a high effective surface area can provide a suitable environment for the interaction of reactants. On the other hand, Ellagic acid (ELA) a bioactive polyphenolic natural compound structurally, is a dilactone of hexahydroxydiphenic acid (HHDP). ELA has the properties of an amphiphilic molecule; structurally, it consists of a planar biphenyl lipophilic moiety bridged by two lactone rings and possessing four hydroxyl groups, which together with lactone groups form a hydrophilic moiety^50^. It seems that the amide bond is formed by the attack of the free -NH_2_ group of APTS molecule and the connection of ellagic acid to SBA-15/APTES.

**Figure 13:**
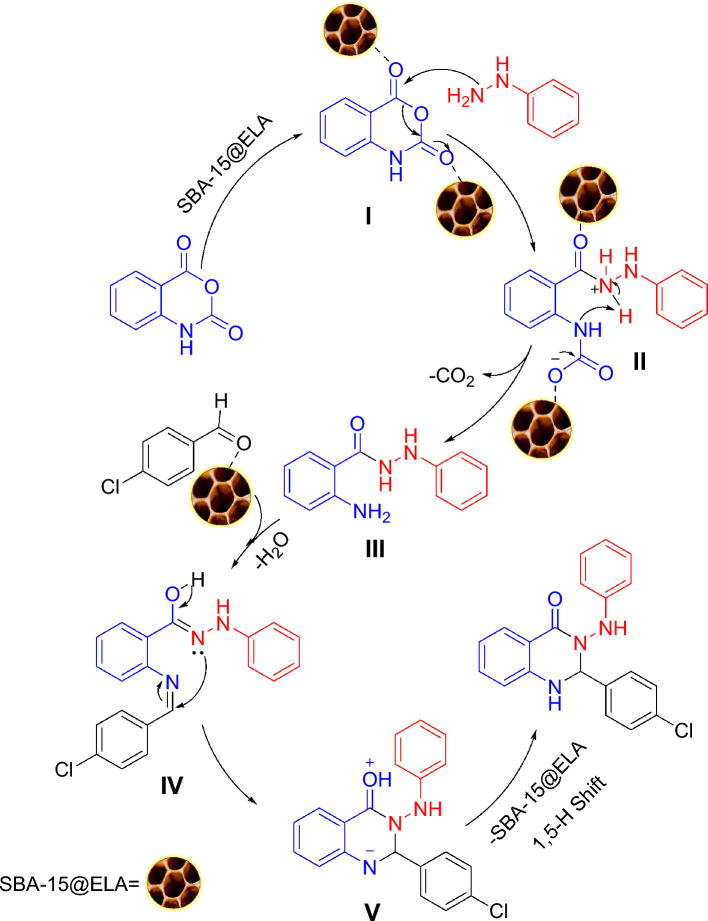
The proposed mechanism for the modified Niementowski reaction in the presence of the SBA-15@ELA as catalyst.

The general scheme of the proposed mechanism for the synthesis of derivatives is shown in Fig. 13. The first step is the interaction of SBA-15@ELA as a catalyst and isatoic anhydride and the activation of its carbonyl groups via hydrogen bonding (**I**). Now, phenyl hydrazine as a nucleophile attacks the carbonyl group of I to produce a reactive intermediate **II**, which gives compound **III** via a decarboxylation reaction. In a separate test, the reaction between isatoic anhydride (**I**) and phenylhydrazine was investigated without SBA-15@ELA. The product **III** (Fig. 13) was formed, although, it was found that in the presence of the catalyst the rate of the reaction was much higher. It means that the catalyst accelerates the reaction, as well as increases the yield. Next, the addition of an aldehyde activated with SBA-15@ELA with the NH_2_ group in **III** gives the imine intermediate **IV**. The intermediate **V** can be prepared by an intermolecular nucleophilic attack of the nitrogen of amide on the activated carbon of the imine, and followed by a 1,5-proton transfer to yield 2,4-oxo-dihydroquinazolinone.

### Reusability of catalyst

**Figure 14:**
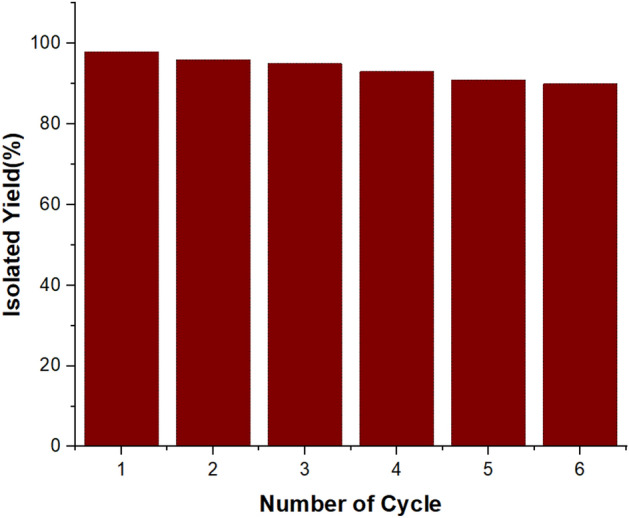
Reusability of SBA-15@ELA for the synthesis of **4a**.

**Figure 15:**
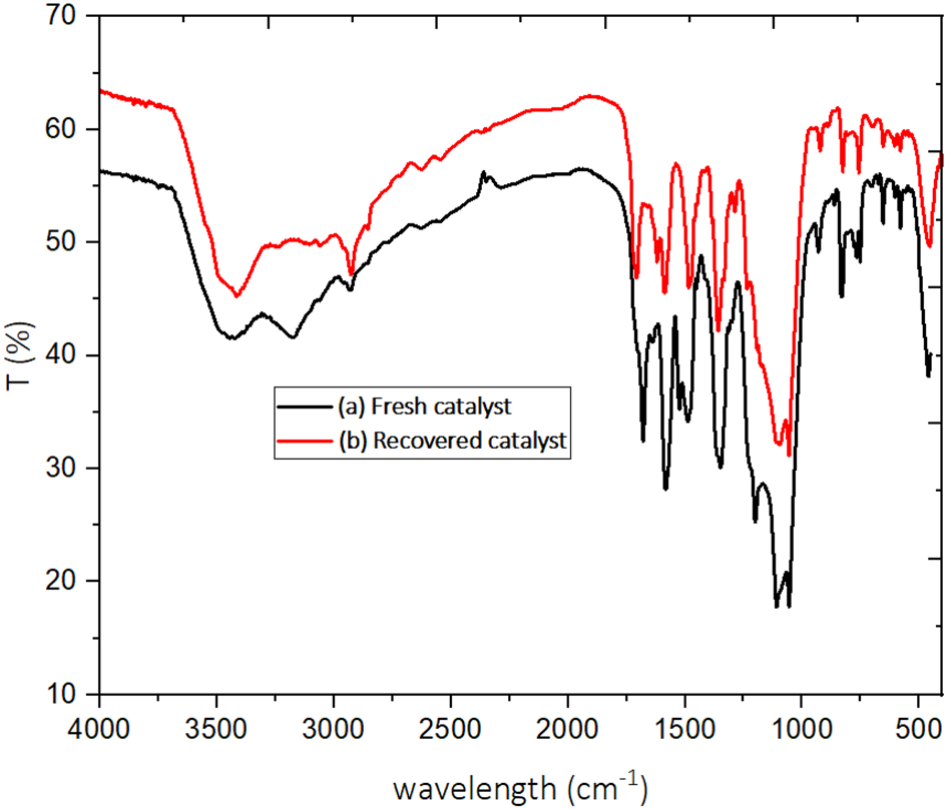
FT-IR spectrum of (**a**) fresh catalyst and (**b**) recovered catalyst.

One of the important topics in green chemistry is the recovery and reusability of catalytic structures in organic reactions. The reusability of SBA-15@ELA as a catalyst in the synthesis of 4-oxo-dihydroquinazolinone (in model reaction for **4a**) was evaluated in several runs. For this purpose, after completion of the reaction, 20 mL hot ethanol was added and stirred. The catalyst was filtered out and washed with ethanol (3 $\times$ 10 mL), then kept in an oven at 70 °C for 10 h and dried to prepare for the next catalytic run. The recovered catalyst was used in a constant amount for the next runs. The results presented in Fig. 14 showed that the recycled catalyst can be used in at least 6 consecutive periods without a significant decrease in its catalytic activity. To prove this, FT-IR spectrum of the fresh catalyst SBA-15@ELA was compared with the recovered catalyst after it has been used six times (Fig. 15). Both spectra show a similar pattern of peaks, suggesting that the fundamental structure of the catalyst remains intact after use.

### Comparison with other catalysts

To show the capability and efficiency of this method and SBA-15@ELA as a suitable catalyst, a comparison is summarized in Table 4 with previous reports for the synthesis of quinazolines. By examining other methods, it was found that this method has a high yield of products in easy reaction conditions.

**Table 4:** Comparison of the catalytic performance of SBA-15@ELA with some other reported catalysts for the synthesis of 4-oxo-dihydroquinazolinone.

Entry	Catalyst	Solvent	Condition	Time (h)	Yield (%)	Ref.
1	Bentonite	H_2_O	Ultrasonic bath/60 °C	0.5	75	^51^
2	Citric acid/Al_2_O_3_	–	Griding	0.1	80	^52^
3	SrFe_12_O_19_ MNPs	–	120 °C	0.25	91	^53^
4	H_3_PO_3_	EtOH	REFLUX	14	95	^28^
5	KAl(SO_4_)_2_·12H_2_O	EtOH	REFLUX	7	91	^54^
6	CeO_2_	EtOH	REFLUX	6	90	^55^
7	SBA-15@ELA	EtOH	REFLUX	6	96	This work

In the newly introduced method, EtOH is used as a green and environmentally friendly solvent instead of hazardous organic solvents. The reaction conditions were straightforward and user-friendly. The SBA-15@ELA functioning as a solid-based heterogeneous catalyst, can be effortlessly isolated from the reaction mixture and reused multiple times. Additionally, the prompt reaction time and high derivative yields further underscore the advantages of this mesoporous nanocatalyst.

## Conclusion

A new mesoporous nanocomposite based on SBA-15 was prepared by modification by APTES and finally functionalization by ellagic acid. This fabricated nanocomposite SBA-15@ELA showed a very good catalytic performance in the synthesis of 4-oxo-dihydroquinazolinone. Various products were obtained with high yields (19 examples with 78–96%), without a complicated synthesis method. The results of TGA analysis showed that this nanocomposite has high thermal stability and could be used for different organic reactions with various temperatures up to 350 °C.

The FESEM images of the mesoporous nanocomposite showed the functionalized SBA-15 porous structure with an average size of about 90 nm, and the TEM images of the nanocomposite showed a regular mesoporous arrangement and a two-dimensional hexagonal honeycomb structure. The key roles of the SBA-15@ELA catalyst are to activate carbonyl groups, enhance their reactivity, and increase the surface area available for the interaction of reactants, thus facilitating the synthesis of the desired product.

### Supplementary Information

Supplementary Information.

## Data Availability

Data is provided within the manuscript or Supplementary information files.
