# Supplementary material for: One-pot synthesis of quinazolinone heterocyclic compounds using functionalized SBA-15 with natural material ellagic acid as a novel nanocatalyst
Source: Sci Rep. 2024 May 16;14:11189. doi: 10.1038/s41598-024-61803-y (PMC11099149; doi:10.1038/s41598-024-61803-y)
Supplement: Supplementary file 1 — Supplementary Information. [file 41598_2024_61803_MOESM1_ESM.pdf]

Supporting information

One-pot synthesis of quinazolinone heterocyclic compounds using functionalized SBA-15 with natural material ellagic acid as a novel nanocatalyst

Nazanin Mohassel Yazdi, Mohammad Reza Naimi-Jamal *

Research Laboratory of Green Organic Synthesis & Polymers, Department of Chemistry, Iran University of Science and Technology, P.O. Box 16846–13114 Tehran, Iran

**Corresponding author E-mail: naimi@iust.ac.ir*

Tel./Fax: (+) 9821–77240289

Table of Content

1. IR-Spectral diagram of selected products…………………………………………….S2-S4
2. ^{1}H and ^{13}C NMR Spectra of selected products……………………………….S5-S8
3. FESEM Images of SBA-15……………………………………………………………………….S9
4. TEM Images of SBA-15…………………………………………………………………………..S9

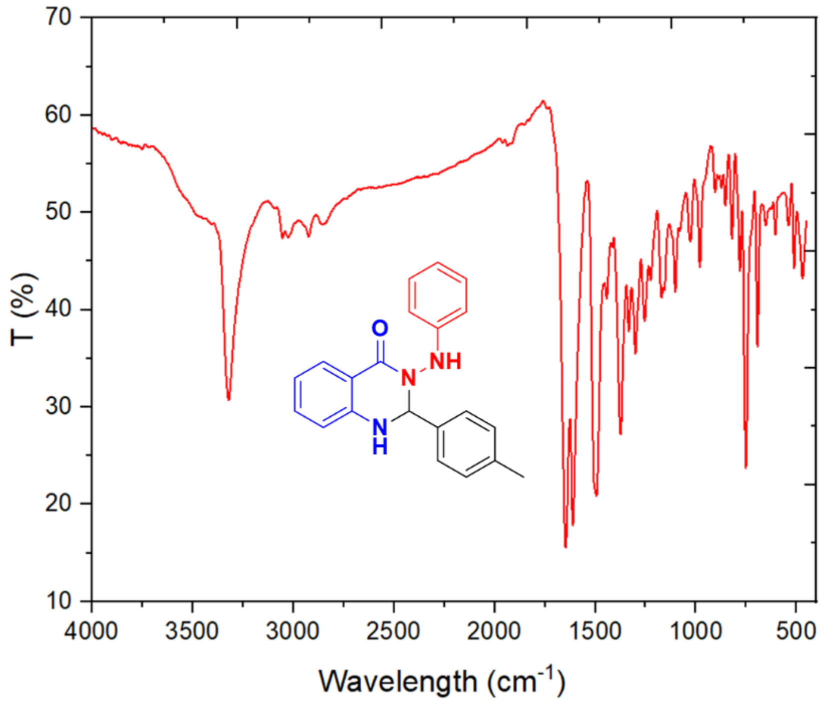

Figure S1. FT-IR spectrum of product (4c)

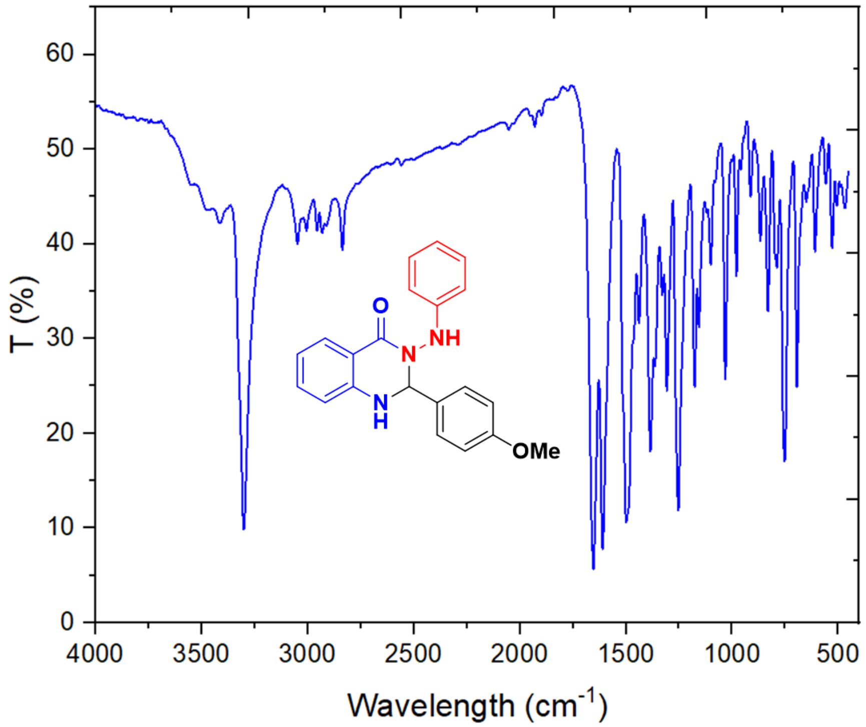

Figure S2. FT-IR spectrum of product (4k)

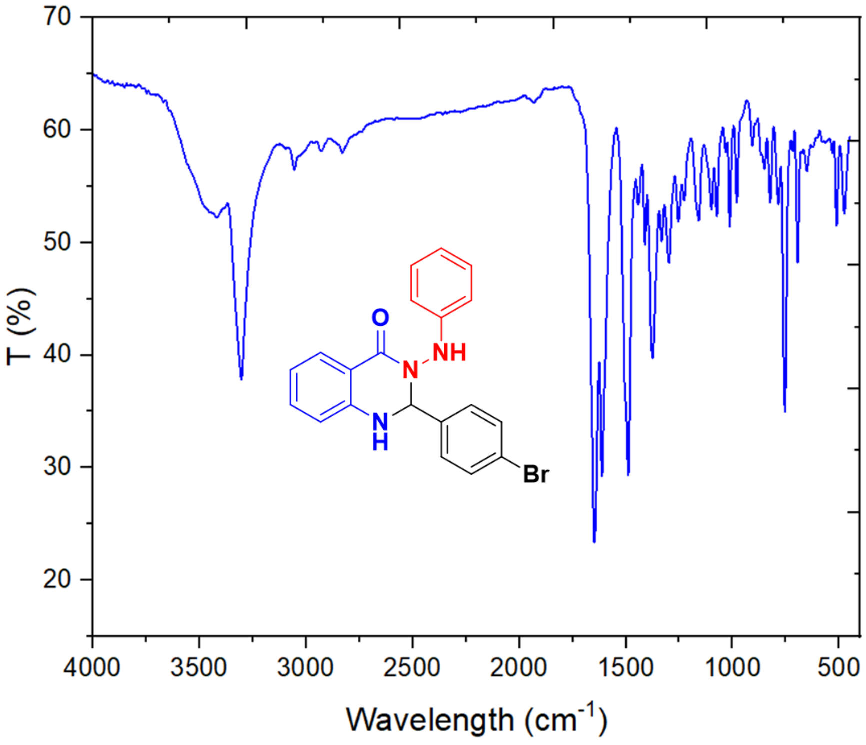

Figure S3. FT-IR spectrum of product **(4d)**

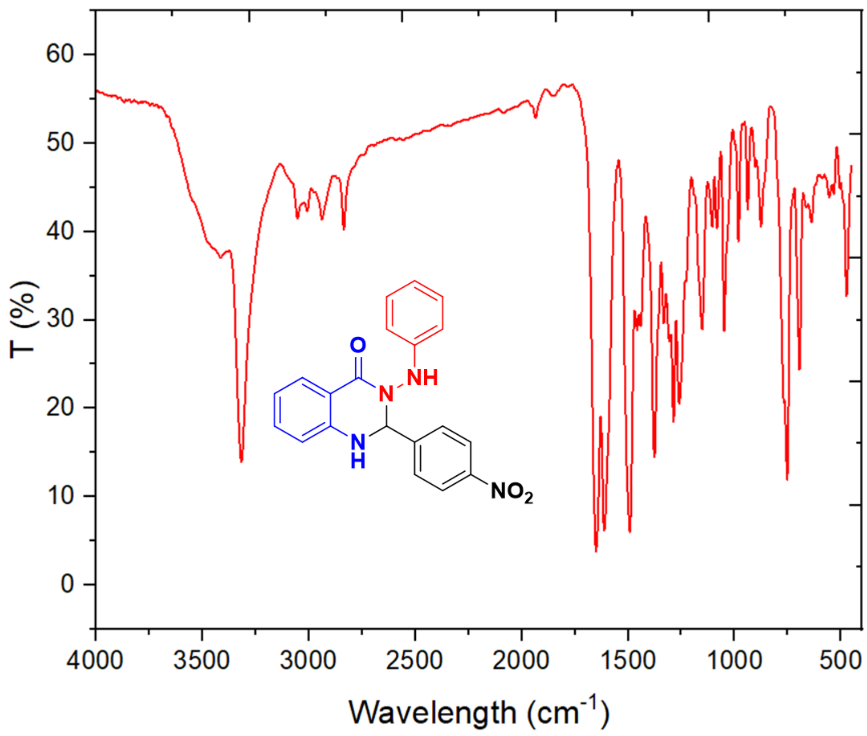

Figure S4. FT-IR spectrum of product **(4b)**

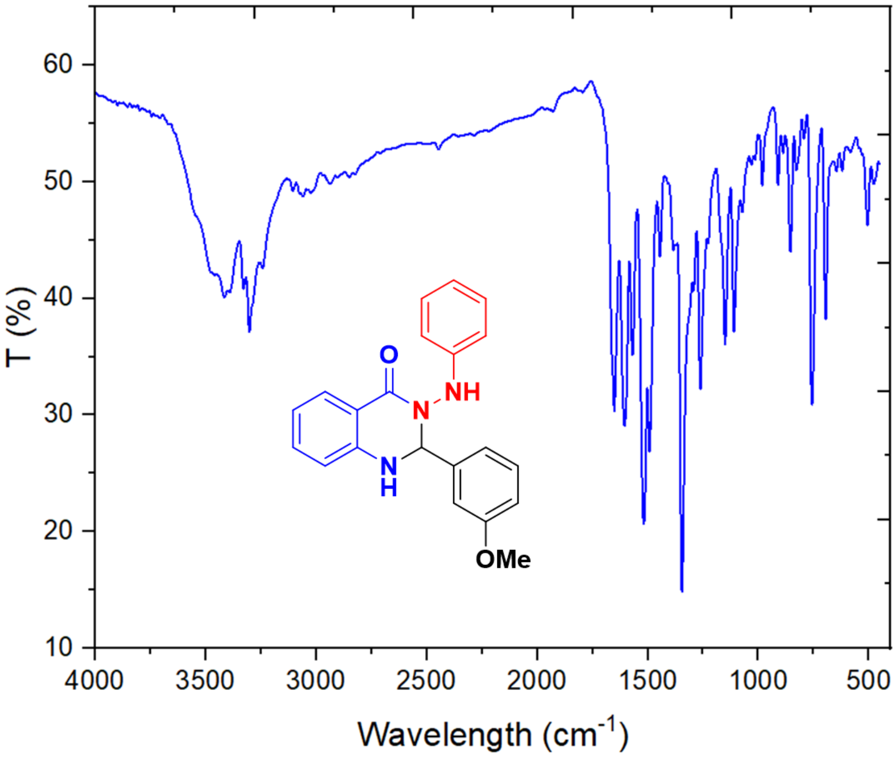

Figure S5. FT-IR spectrum of product (4l)

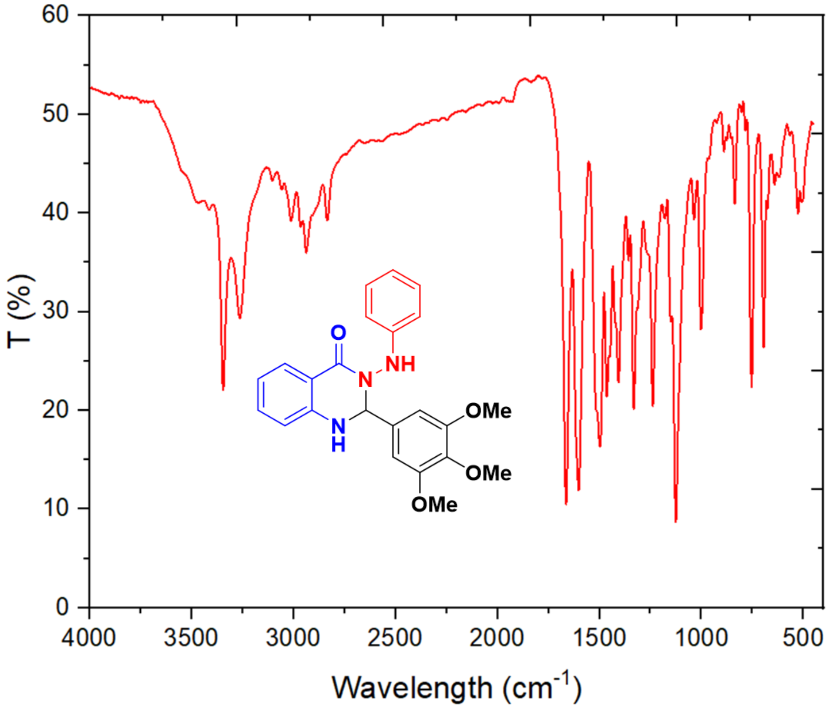

Figure S6. FT-IR spectrum of product (4r)

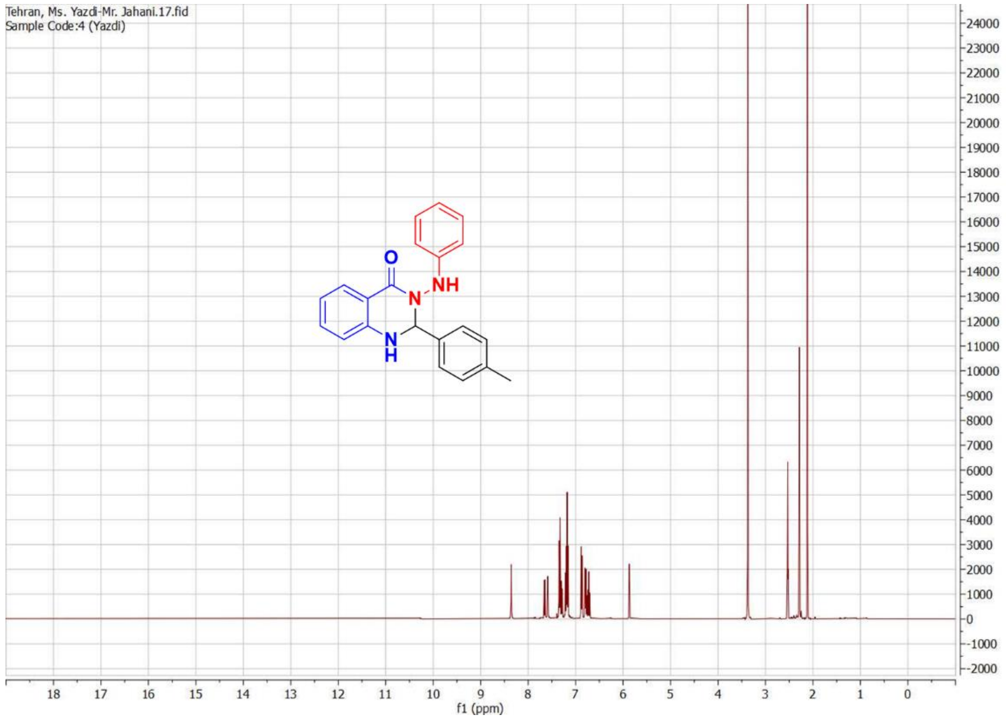

Figure S7. ^{1}H NMR (400 MHz, DMSO-d^{6}, 25 °C) spectrum of product **(4c)**

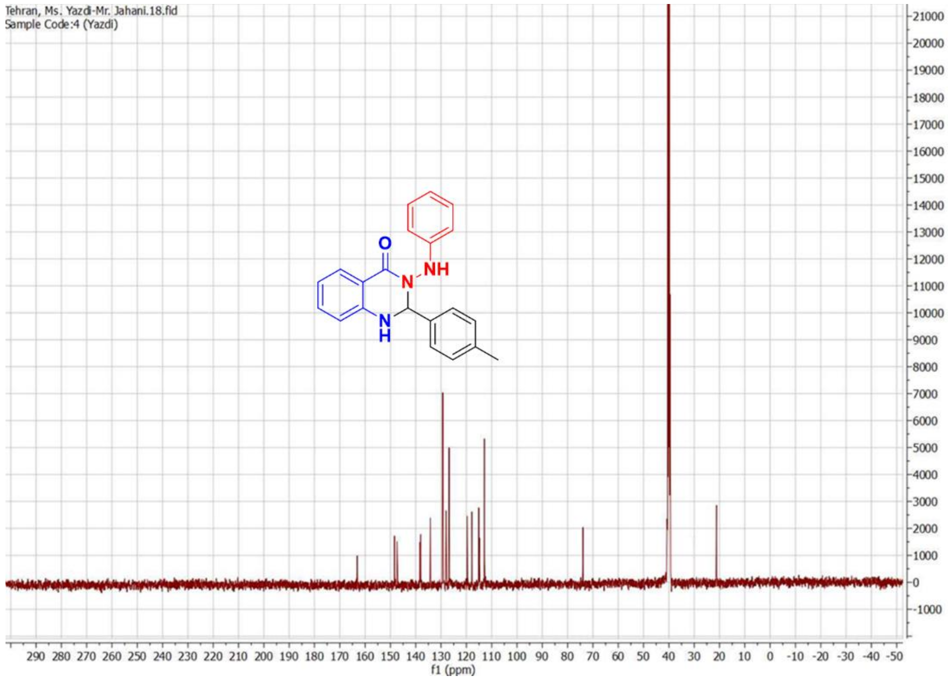

Figure S8. ^{13}C NMR (100 MHz, DMSO-d^{6}, 25 °C) spectrum of product **(4c)**

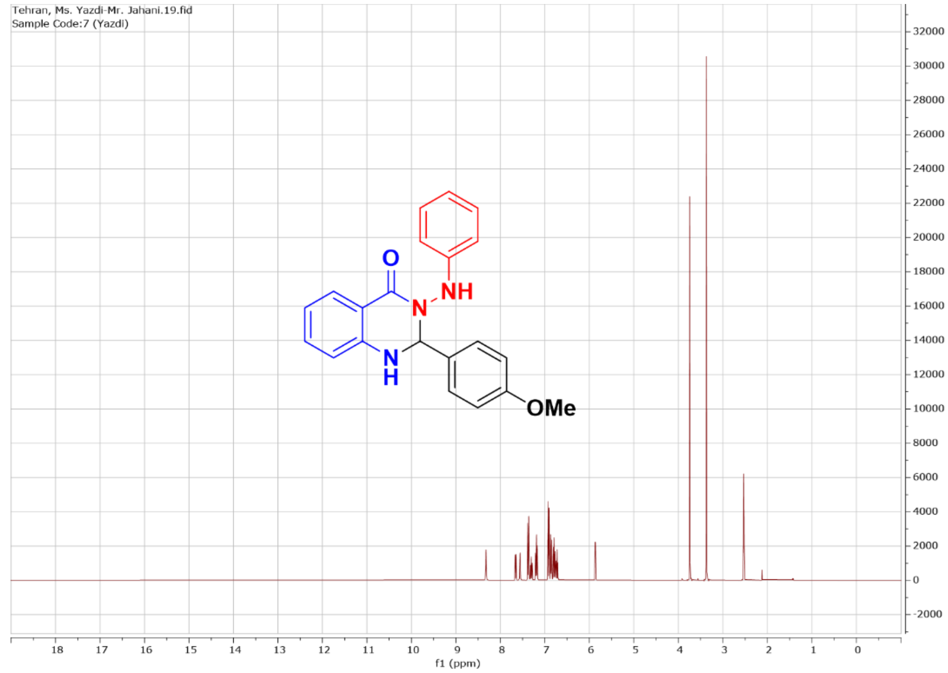

Figure S9. ^{1}H NMR (400 MHz, DMSO-d[6], 25 °C) spectrum of product **(4k)**

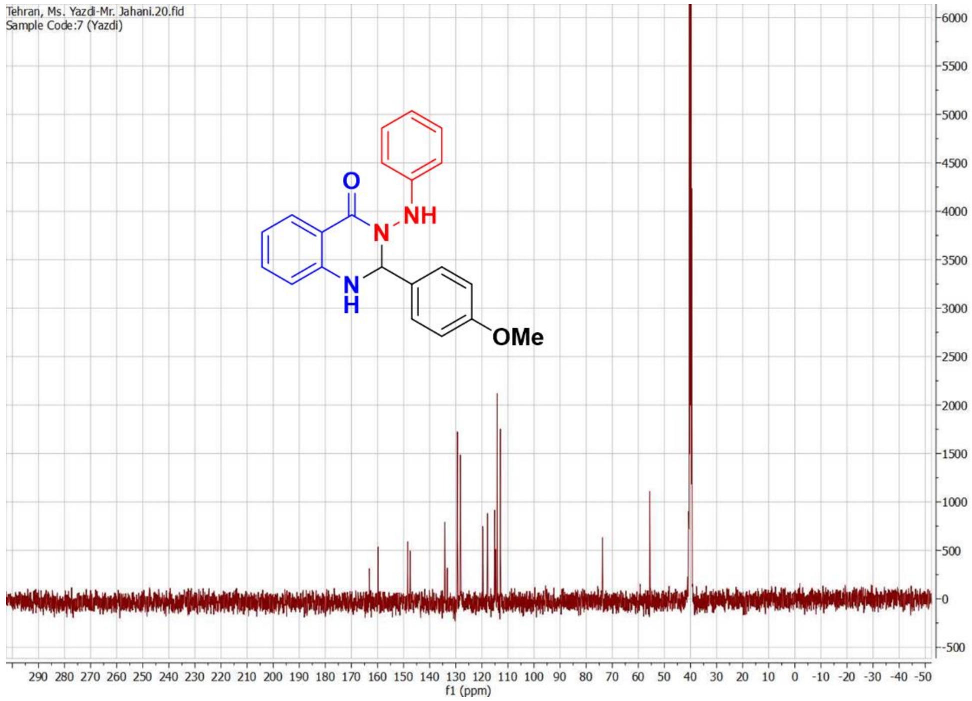

Figure S10. ^{13}C NMR (100 MHz, DMSO-d[6], 25 °C) spectrum of product **(4k)**

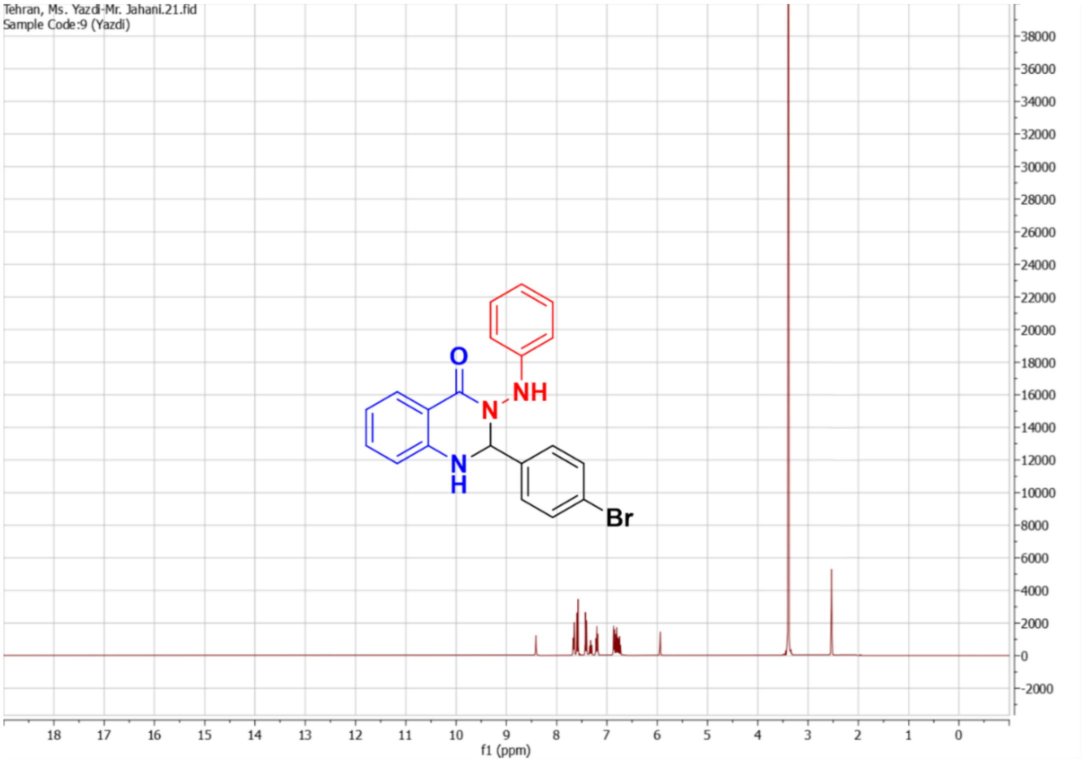

Figure S11. ^{1}H NMR (400 MHz, DMSO-d^{6}, 25 °C) spectrum of product **(4d)**

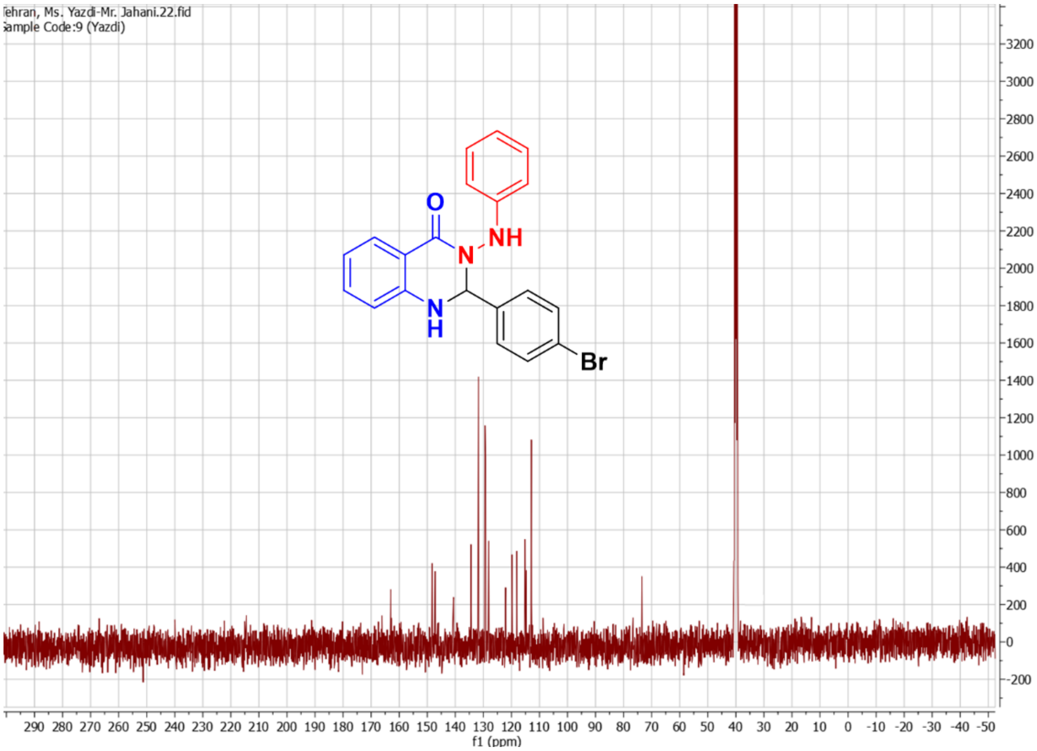

Figure S12. ^{13}C NMR (100 MHz, DMSO-d^{6}, 25 °C) spectrum of product **(4d)**

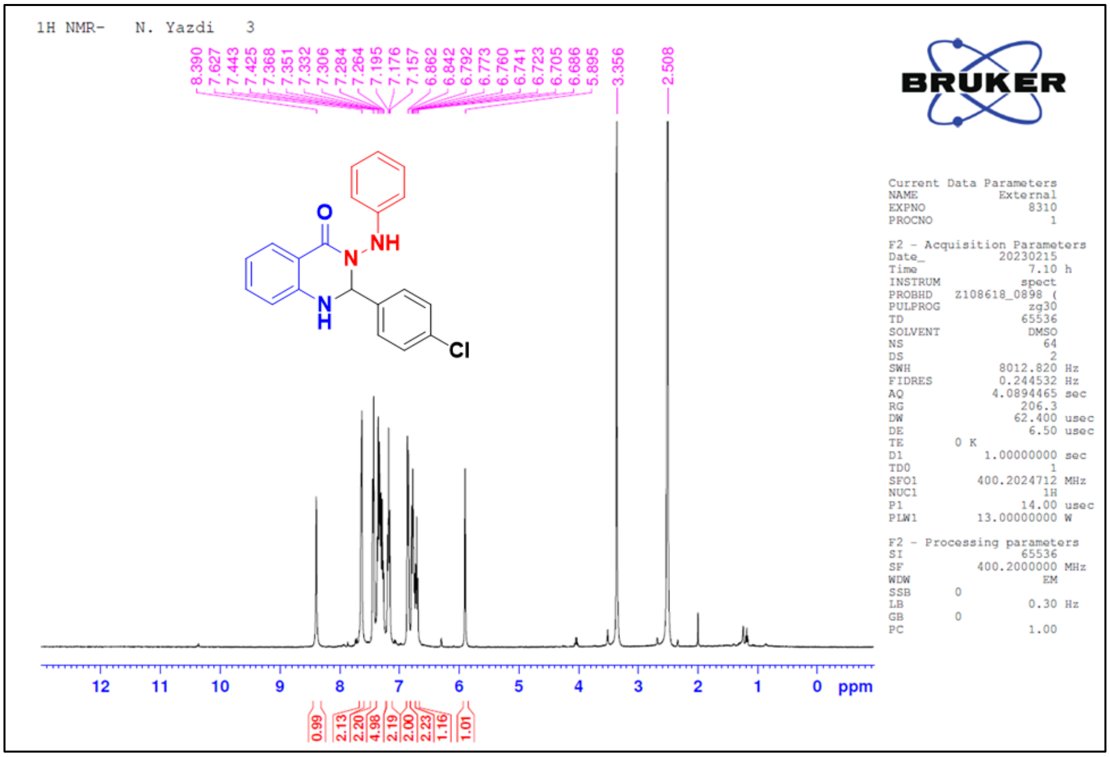

Figure S13. ^{1}H NMR (400 MHz, DMSO-d^{6}, 25 °C) spectrum of product **(4i)**

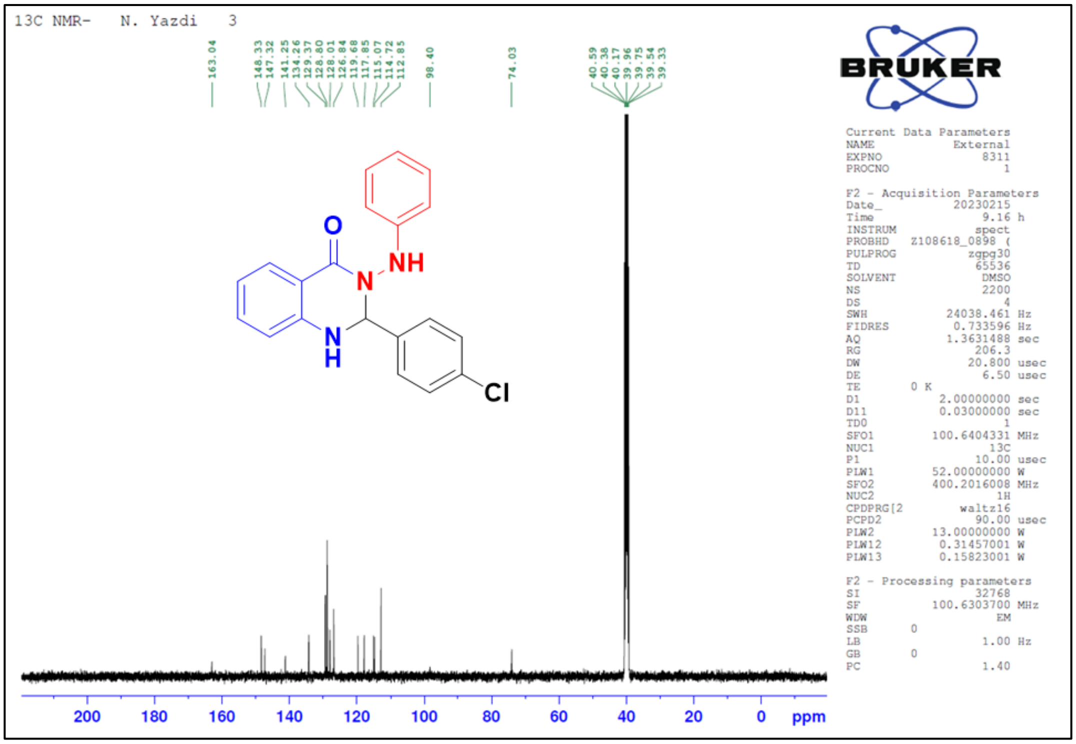

Figure S14. ^{13}C NMR (100 MHz, DMSO-d^{6}, 25 °C) spectrum of product **(4i)**

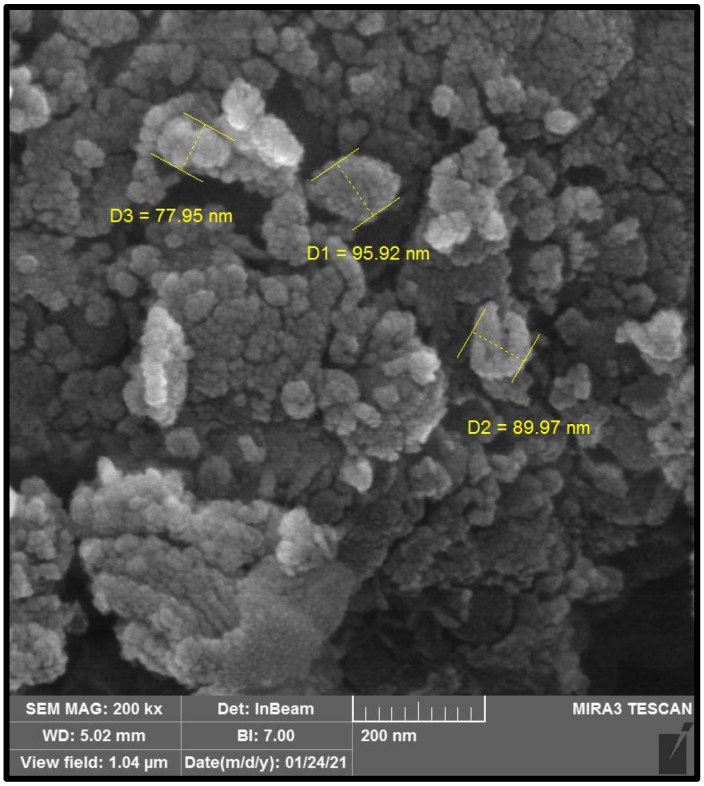

Figure S15. FESEM image of **SBA-15**

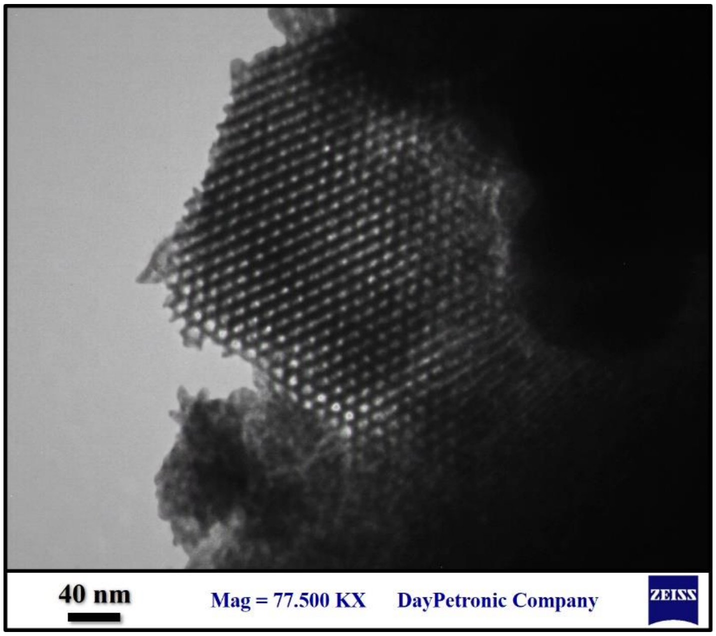

Figure S16. TEM image of **SBA-15**
